# Salivary concentrations of *Streptococcus mutans* and *Lactobacilli* during an orthodontic treatment. An observational study comparing fixed and removable orthodontic appliances

**DOI:** 10.1002/cre2.261

**Published:** 2019-12-03

**Authors:** Stefano Mummolo, Marco Tieri, Alessandro Nota, Silvia Caruso, Atanaz Darvizeh, Francesca Albani, Roberto Gatto, Giuseppe Marzo, Enrico Marchetti, Vincenzo Quinzi, Simona Tecco

**Affiliations:** ^1^ Department of Life, Health and Environmental Sciences University of L'Aquila L'Aquila Italy; ^2^ Department of Medical, Oral and Biotechnological Sciences University “G. D'Annunzio of Chieti‐Pescara Chieti Italy; ^3^ Dental School, Vita‐Salute San Raffaele University and I.R.C.C.S. San Raffaele Hospital Milan Italy

**Keywords:** Clear aligners, Dental Plaque Index, Fixed orthodontic appliances, *Lactobacilli*, Microbial colony count, Oral Hygiene, Orthodontics, Removable orthodontic appliances, Removable positioner, Saliva, *Streptococcus mutans*

## Abstract

**Aim:**

This study aimed to investigate salivary concentrations of *Streptococcus mutans* (*S. mutans*) and some *Lactobacilli*, and plaque index (PI) in patients wearing fixed versus removable orthodontic appliances.

**Methods:**

A sample of 90 orthodontic patients (56 males and 34 females) was included in the study: 30 subjects (aged 21.5±1.5 years) were treated with removable clear aligners (CA), while for other 30 cases (aged 23.3±1.6 years) a fixed multibrackets appliance (MB) were utilized, and 30 patients (aged 18.2 ±1.5 years) wearied a removable positioner (RP). Salivary concentrations of *S. mutans* and *Lactobacilli* and PI were evaluated prior to start of the orthodontic treatment, after 3 months and 6 months.

**Results:**

After 6 months, 40% of MB patients (12 subjects over 30) showed a concentration of *S. mutans* associated to high risk of developing tooth decay (CFU/ml>10^5^), differently from participants wearing removable appliances (odds ratio = 5.05; 95% C.I. = 1.72‐14.78; chi‐square = 9.64; p = 0.0019). The same trens was observed for the concentration of *Lactobacilli* (odds ratio = 4.33; 95% C.I. = 1.53‐12.3; chi‐square = 8.229; p = 0.004).

In addition, over the duration of the study, CA patients maintained PI at 0 level, while MB patients experienced a statistically significant increasing trend of PI over time, and their PI became clinically/statistically relevant after 6 months, respect to CA and RP patients.

**Conclusions:**

Comparing all the data, while, after 6 months, only about 10% of CA patients and 13.3% of RP patients achieved a microbial colonization which may lead to high risk of caries development, about 40% of MB patients ‐ and 20% after 3 months ‐ showed a high level of vulnerability to developing caries, which require additional strategies for plaque control and microbial colonization to be employed.

## 1 INTRODUCTION

Two literature reviews (Freitas, Marquezan, Nojima, Alviano, & Maia, 2014) (Lucchese, Bondemark, Marcolina, & Manuelli, 2018) show that there is moderate‐to‐high evidence that orthodontic appliances are able to significantly influence the concentration of oral microbiota, causing an alteration of the quantity of *Streptococcus mutans* (*S. mutans*) and *Lactobacilli* that can basically affect the process of dental caries and tooth enamel demineralization, due to their acid production and tooth adhesive properties. This statement is confirmed both for removable (Mummolo et al., 2014) and fixed (Mummolo et al., 2013) orthodontic appliances.

From a clinical point of view, as a probable consequence of the changes in microbiota, caries incidence increases during orthodontic treatment with multibrackets appliances (MB), as well as the occurrence of white spot lesions, which range, among orthodontic patients, from 2% to 97% (Migliorati et al., 2015; Mummolo, Nota, De Felice, et al., 2018). On the contrary, it was observed that removable orthodontic appliances have less impact on the oral microbiota than fixed ones (Mummolo et al., 2014), but literature still lacks data on clear aligners (CA), a removable appliance that has become highly demanded as an alternative to MB, owing to its desirable characteristics providing aesthetic and comfortability. (Brignardello‐Petersen, 2019)

The attention on clear aligners has ultimately increased over time, mostly because they are often used to manage pre‐prosthetic clinical cases (Dinoi et al., 2015), also affected by temporo‐mandibular joint diseases, (Tecco, Festa, Salini, Epifania, & D'Attilio, 2004) or adult patients for which a molar distalization is required, owing a great attention to the periodontal health status (Caruso et al., 2019) But, also in children, this type of removable appliance has been increasingly used, for example, to manage clinical cases with a single tooth anterior crossbite, to prevent further progress of periodontal diseases. (Meuli, Tecco, Nota, Gatto, & Caruso, 2018) (Silvestrini‐Biavati et al., 2013)

Furthermore, considering previous observations for removable appliances (Lucchese et al., 2018) (Mummolo et al., 2014), there exist a general clinical trend to prefer CA to maintain a more satisfactory level of oral hygiene during the orthodontic treatment. It can be justified that to obtain a better periodontal health the CA appliance can be more desirable than MB appliances. (Levrini et al., 2015)

Due to the lack of empirical data this study aims to provide a solid base for assessment of a preferable trend that rempvable appliance, in particular CA appliance, can provide a more suitable clinical treatment while reducing the concentrations of *S. mutans* and *Lactobacilli* in saliva.

In addition, the plaque index (PI) was also evaluated, because as evidence exists for salivary bacterial concentrations being an acceptable approximate representation of dental plaque, they aren't nonetheless an indirect measure of the bacterial threat on the teeth. Thus, the recording of PI parallel with the count of cariogenic species in the saliva could be a validation of the clinical conclusions derived from the data.

### 1.1 Study population and methodology

This is an observational controlled study, aimed to investigate salivary concentrations of *S. mutans* and *Lactobacilli*, and PI in patients wearing fixed *versus* removable orthodontic appliances. The participants were selected from a population of young adult patients who were going to be treated for their malocclusion in a dental clinic in the geographical region of Abruzzo (Central Italy).

A total sample of 90 patients were included.

For 30 of them, the treatment plan included clear aligners (CA) (Invisalign, Align Technology, Santa Clara, CA, USA), while other 30 subjects were going to be treated with a multibrackets fixed orthodontic appliance (MB) (Damon Q2, Ormco, Washington, DC, USA); the other 30 participants were treated with removable positions (RP) (Occlus‐o‐Guide, Sweden&Martina, Padova, Italy).

Demographic data of the sample are described in table 1. The matching criteria were confirmed since no significant difference was detected in gender distribution and mean age among the participants of the three groups.

**Table 1:** Demographic data in the whole sample

	Clear aligners (CA group)	Multibrackets appliance (MB group)	Removable Positioner (RP group)
**Gender**	18 M and 12 F	22 M and 12 F	16 M and 10 F
**Numerosity**	30	30	30
**Age (mean** ± **sd)**	21.5 ± 1.5 years	23.3 ± 1.6 years	18.2 ± 1.5

The orthodontic technique adopted for the treatment of each subject has been already chosen for each partecipant by the expert orthodontists, prior to the beginning of this research project.

The selection of participants was made on the bases of the following inclusion criteria: permanent dentition, adult age, complete dentition, and a malocclusion characterized by Angle class I, with low‐middle level of crowding (the subjects did not require any orthodontic teeth extraction). The following parameters were taken as exclusion criteria: chronic periodontitis, presence of prosthetic rehabilitations, presence of endodontically treated teeth, history of high grade gingivitis, poor oral hygiene, initial Plaque index (PI) and Bleeding index (BI). The enrollment of the participants was done from January 2016 to April 2018.

All the participants were treated by two expert orthodontists (author F.A. and author S.C.), who exclusively practice the branch of orthodontics for more than 5 years.

Data collection and follow‐up of the subjects were performed in the following way. A proper informed consent form was signed by each subject during one of the preliminary appointments before beginning the actual treatment. To motivate the participants, a free oral hygiene session (with instructions) was offered. Furthermore, subsequent follow‐up visits included in the present protocol were arranged free of charge. This clinical protocol was in accordance with the ethical standards reported in the Declaration of Helsinki of 1975, and the study was ethically approved by the Ethical Committee of the University of L'Aquila (Document DR206/2013).

As the home oral hygiene habits could be considered as a potential confounder, a few days before the beginning of the observational period, a professional oral hygiene procedure was performed on each participants, and accurate oral hygiene instructions were given to each subject to be employed at home. Then, at the day scheduled for the beginning of the orthodontic treatment a salivary sample was taken, and the PI was recorded, from each subject preceding to the bonding procedure. Other salivary samples and recordings of PI were taken after periods of 3 and 6 months. All the salivary samples were taken by the same operator.

The salivary samples were analyzed by *CRT® bacteria* (Ivoclar Vivadent Clinical, Schaan, Liechtenstein). The *CRT® bacteria* was employed for the bacterial count, as previously published. (Mummolo et al., 2013; Mummolo, Nota, Caruso, et al., 2018)

*CRT® bacteria* was used to determine the *S. mutans* and *Lactobacilli* count in saliva by means of selective culture media. The dentist and skilled personnel professionally conduct the test. Findings of 10^5^ CFU or more of *Lactobacilli* and *S.mutans* per ml saliva indicate a high caries risk. Leaving the test vial inside an incubator for an additional day or two does not influence the number of CFUs. The preparation of samples and incubation were carried out according to the step‐by‐step procedure as it was described in its instruction brochure. This test only determines whether or not *S. mutans* are present in dental saliva.

The *CRT® bacteria* can be considered a comprehensive test, whose main benefits are to determine the caries risk status, to create the basis for target treatment and individualized check‐up intervals for the long‐term maintenance of oral health. This chair‐side method is highly specific and sensitive for *S. mutans (*Sánchez‐García et al., 2008 *)* and its only limitation is that at least 48 hours are require for detection of *S. mutans.*

At each of the follow‐up appointments (at the beginning, after 3 months, and after 6 months), firstly, the PI was recorded. Then, the patient was asked to chew a stimulant paraffin tablet for 30 seconds, then the secreted saliva was collected in a glass tube and used for bacterial count through the *CRT® bacteria.* The saliva was placed in culture media (agar). A NaHCO_3_ tablet was added to stimulate the growth of bacteria and each culture was placed in an incubator at 35‐37 °C for 48 hours. *S.mutans* colonies appeared visible as small blue colonies with a diameter < 1mm on blue agar, while white color *Lactobacilli* colonies were detected on transparent agar. The presence of a bacterial count higher than 10^5^ CFU/ml of saliva indicates a high risk of developing caries (cut‐off value for the high risk). Thus, in this study, subjects were dichotomized as *S. mutans* and *Lactobacilli* CFU > or < 10^5^ CFU/ml, that is considered the cut‐off value for the high risk. (Messer, 2000) (Kõll‐Klais, Mändar, Leibur, & Kjaeldgaard, 2004) (Mummolo et al., 2013) (Mummolo et al., 2014)

#### 1.1.1 Data analyses

To avoid bias, the data were analyzed by operators who were blind to the fact that each collected data belongs to which group.

The data about the microbiota were examined considering the number of subjects (and percentage) with CFU/ml ≥ or < the cut‐off value (*i.e.* 10^5^ CFU/ml). These percentages were compared over time at the beginning of the treatment, after 3 months and after 6 months from the beginning, and differences among the three groups were investigated using the Chi‐square test.

PI was handles as a continuous variable, and recorded through 0, 1, 2, and 3 values. Descriptive statistics included mean and standard deviation, and differences among the two groups were tested by the ANOVA statistics, and post‐hoc comparisons.

For all the analysis the p value was set at 0.05.

## 2 RESULTS

All the participants completed the study, without any adverse event, and there weren't missing data.

Table 2 shows the percentage of patients with *S. mutans* CFU/ml >10^5^ over time in the three groups. In the CA group, the number of patients with CFU/ml >10^5^ slightly increased after 6 months, without statistical relevance. A same trend was observed in patients wearing RP appliances. In the MB group, it increased progressively over time, with a statistically significant difference from beginning to 3 months, and from beginning to 6 months. The differences among the groups were statistically significant at 3 months and at 6 months.

**Table 2:** Number (and percentage) of patients with S. Mutans or Lactobacilli CFU >10^5^, at t0, t1 and t2, with intra‐group and between‐groups differences

S.mutans						
	t0 Initial	t1 After 3 months	t2 After 6 months	t0 versus t1 comparison	t0 versus t2 comparison	t1 versus t2 comparison
**Clear aligners (CA group)**	0 over 30 (0%)	0 over 30 (0%)	3 over 30 (10%)	n.s.	n.s.	n.s.
**Multibrackets appliance (MB group)**	0 over 30 (0%)	6 over 30 (20%)	12 over 30 (40%)	Chi‐square = 6.66 p=0.009	Chi‐square = 15 p=0.000	n.s.
**Removable Positioner (RP group)**	0 over 30 (0%)	2 over 30 (6.6%)	4 over 30 (13.3%)	n.s.	n.s.	n.s.
**CA group versus MB group** **Comparison**	n.s.	Chi‐square = 6.66 p=0.009	Chi‐square = 7.2 p=0.007			
**RP group versus MB group** **Comparison**	n.s.	n.s.	Chi‐square = 5.45 p=0.019			
**CA group versus RP group** **Comparison**	n.s.	n.s.	n.s.			

*Lactobacilli*						
	t0 Initial	t1 After 3 months	t2 After 6 months	t0 versus t1 comparison	t0 versus t2 comparison	t1 versus t2 comparison
**Clear aligners (CA group)**	0 over 30 (0%)	1 over 30 (3.3%)	4 over 30 (13.3%)	n.s.	n.s.	n.s.
**Multibrackets appliance (MB group)**	0 over 30 (0%)	8 over 30 (26.6%)	12 over 30 (40%)	Chi‐square = 8.08 p= 0.004	Chi‐square = 15 p=0.000	n.s.
**Removable Positioner (RP group)**	0 over 30 (0%)	2 over 30 (6.6%)	4 over 30 (13.3%)	n.s.	n.s.	n.s.
**CA group versus MB group** **Comparison**	n.s.	Chi‐square = 6.40 p=0.01	Chi‐square = 5.45 p=0.019			
**RP group versus MB group** **Comparison**	n.s.	n.s.	Chi‐square = 5.45 p=0.019			
**CA group versus RP group** **Comparison**	n.s.	n.s.	n.s.			

n.s. = not significant

At 6 months, MB patients manifested an odds ratio of 5.05 (95% C.I. = 1.72‐14.78; chi‐square = 9.64; p = 0.0019), in respect to the participants wearing removable appliances, which clearly indicates that MB subjects after 6 months of treatment are at higher risk of developing caries, due to the elevated concentrations of *S. mutans.*

Table 2 also reports the percentages of subjects who showed the *Lactobacilli* CFU/ml >10^5^ (cut‐off value). In the MB group, it increased progressively over time, with a statistically significant difference from beginning to 3 months and to 6 months.

After 6 months of treatment, patients wearing MB showed an odds ratio of 4.33 (95% C.I. = 1.53‐12.3; chi‐square = 8.229; p = 0.004) respect CA patients and RP patients, to develop salivary concentrations of *Lactobacilli* CFU/ml >10^5^

Table 3 reports descriptive statistic (mean ± standard deviation) for PI over time. In the CA group PI remained 0 all over the follow‐up period, while a progressive statistically significant increase over time was observed in MB patients, with significant differences in comparison with CA patients at 3‐6 months, and with RP patients at 6 months.

**Table 3:** Plaque Index (mean and standard deviation) in the three groups at t0, t1 and t2, with intra‐group and between groups differences

	t0 Initial	t1 After 3 months	t2 After 6 months	t0 versus t1 comparison	t0 versus t2 comparison	t1 versus t2 comparison
**Clear aligners (CA group)**	0	0	0	n.s.	n.s.	n.s.
**Multibrackets appliance (MB group)**	0	0.7 ± 0.59	1.3 ± 0.46	p = 0.00	p = 0.00	p = 0.00
**Removable Positioner (RP group)**	0	0.37 ± 0.48	0.97± 0.8	n.s.	p = 0.00	n.s.
**CA group versus MB group** **comparison**	n.s.	p = 0.001	p = 0.00			
**RP group versus MB group** **Comparison**	n.s.	n.s.	p = 0.00			
**CA group versus RP group** **Comparison**	n.s.	n.s.	n.s.			

n.s. = not significant

## 3 DISCUSSION

This study aimed to investigate salivary consentrations of *S. mutans* and *Lactobacilli*, and PI in patients wearing removable (CA or RP) or fixed (MB) orthodontic appliances.

A different trend in bacterial colonization among the three groups was observed, with statistically significant differences among the fixed orthodontic appliance *versus* the removable ones.

All the CA patients showed *S. mutans* and *Lactobacilli* concentration lower than the critical value, which can lead to develop caries (i.e. 10^5^/ml), at the beginning of the study and at 3 months. While patients with a fixed appliance (MB) showed a progressive increase over time from the beginning of the study to 3 months (p<0.01) and at 6 months, when the 40% of patients (12 patients over 30) showed concentrations at high risk of developing of tooth decay (i.e. 10^5^/ml).

After 6 months, 40% of MB patients, versus 10‐13.3% of CA and RP patients, respectively, showed concentrations at high risk of developing of tooth decay (i.e. 10^5^/ml), with a statistically significant difference.

As seen, the count of *Lactobacilli* colonies showed a trend similar to *S. mutans* (table 2) in all the three groups.

The observed bacteria colonization suggests that the considered removable orthodontic appliances (CA and RP) do not increase the bacterial growth in the saliva, and, consequently, the caries risk, in about the 87.7% (for RP) and 90% (for CA) of the treated subjects, even after 6 months of treatment. While at the authors’ knowledge there are no previous studies in literature that performed a microbiological analysis of salivary bacteria in patients using CA, a recent prospective study, performed by quantitative polymerase chain reaction, found no differences in the identification of *S. mutans*, and *Lactobacillus acidophilus (L. acidophilus)*, in the saliva of patients wearing thermoplastic retainers compared with subjects treated with fixed appliances. But in that study no quantitative analysis was possible, as almost no *L. acidophilus* was identified in the collected samples. (Sifakakis et al., 2018)

From this study it seems that there is no relevant difference between the considered two removable devices. CA as well as RP do not cause increases in bacterial colonization of the saliva in the first 6 months of treatment, when it becomes advisable a significant difference with MB appliance.

Also, it results from the present data, that PI remained 0 in CA patients until 6 months, while MB patients experienced a statistically significant increasing trend, with statistically remarkable differences respect to the other groups after 3 months and 6 months of treatment.

This last observation suggests that CA and RP allow the maintenance of a better oral hygiene level, respect to MB. This observation agrees with previous data from literature, indicating that teenagers treated with CA show a notably lower plaque index than those treated with MB, even at a follow up of 12 months. (Abbate et al., 2015) In addition, a systematic review pointed out, with a level of moderate evidence, that periodontal health indexes are also considerably better with CA respect to MB. (Rossini, Parrini, Castroflorio, Deregibus, & Debernardi, 2015)

From a clinical point of view, comparing all the data, it can be stated that only about 10% of CA and 13.3% of RP patients may reach the require microbial concentrations for being in the high risk of developing tooth decay (i.e. 10^5^/ml) after 6 months of treatment, with a stable plaque control, differently from MB patients. The result obtained for PI can be regarded as a validation of the conclusions derived from the microbiological data. The present results give an acceptable representation of bacterial concentration in dental plaque, but can be only an indirect measure of bacterial threat to the teeth.

The maintenance of a better macroscopically (plaque index) and microscopically (*S.mutans* and *Lactobacilli* CFU) oral hygiene levels in patients with removable appliances, should be related to the absence of fixed retentive surfaces on the patient's teeth and with the consequent facilitation of oral hygiene procedures, which is the same of untreated subject. (Mummolo et al., 2013) While in about 40% of MB patients there exists an increased risk of dental caries and demineralization after 6 months from starting the treatment (and for 20% just after 3 months), and additional strategies for plaque control must be triggered, the use of CA and RP seems to significantly reduces this need to about 10% of the subjects treated with CA and 13.3% of the subjects treated with RP. The necessity of employing additional strategies in patients with microbial salivary concentrations, who are facing the high risk of developing tooth decay (i.e. 10^5^/ml) could be the reason to use disinfectant and mineralizing substances. (D'Ercole, Martinelli, & Tripodi, 2014) An additional aid for these patients can be provided by the use of food, like some yoghurt that can positively influence the oral ecosystem. (Ferrazzano et al., 2017)

This study was subjected to some limitations, for instance only patients with Angle class I malocclusion with low/mild level of crowding were included, and consequently it wasn't possible to analyze the type of malocclusion as a confounding factor, therefore the present results can not be generalized for other type of malocclusions with more severe level of crowding. In addition, smoking patients were not included, while it is known that salivary immunoglobulin and periodontal status can be affected by the smoking habit, potentially influencing bacterial colonization. (Giuca, Pasini, Tecco, Giuca, & Marzo, 2014)

Furthermore, no certain data were included about the compliant of the participants regarding the quality of home oral hygiene, although proper instructions were given to all the participants. In addition, another limitation of the present study was the use of a paraffin wax to stimulate salivary flow, which may have slightly modified the results by detaching bacteria from surfaces.

## 4 CONCLUSIONS

Comparing all the data, it can be stated that only about 10% of CA patients and 13.3% of RP patients achieve microbiota salivary concentrations and they are subjected to high risk of developing caries (i.e. 10^5^/ml) after 6 months of treatment, compared to MB patients, for which about 40% of cases are highly vulnerable to developing caries, and require additional strategies for plaque control and microbial colonization must be controlled just after the first 6 months of treatment.

### Clinical Relevance

In general, it was observed that removable appliances have less impact on the oral microbiota than fixed ones, but literature still lacks data on clear aligners (CA), an appliance that has become an increasingly applied treatment of choice because of the great number of patients that desire aesthetic and comfortable alternatives to multibracket appliances (MB).

### Principal findings

After 6 months of treatment, MB patients manifested an odds ratio of 5.05 (95% C.I. = 1.72‐14.78; chi‐square = 9.64; p = 0.0019) to show concentrations of *S. mutans* at high risk of developing teeth decay, respect to CA and RP patients; and an odds ratio of 4.33 (95% C.I. = 1.53‐12.3; chi‐square = 8.229; p = 0.004) to develop concentrations of *Lactobacilli* at high risk of developing teeth decay. Patients with CA maintained plaque index at level 0 all over time, while patients wearing MB orthodontic appliances experienced a statistically significant increase in the plaque index over time. The differences in the plaque index became clinically/statistically relevant after 3 months respect to CA appliance, and after 6 months respect to RP patients.

**Practical implications:** the clinician must be aware that different preventive protocols of oral diseases should be applied to orthodontic patients wearing CA, compared to patients wearing MB, because only about 10% of CA patients and 13.3% of RP patients achieve microbiota concentrations at high risk of developing teeth decay after 6 months of treatment, differently from MB patients, for which about 40% of cases are highly vulnerable to developing caries, and require additional strategies for plaque control and microbial colonization must be controlled just after the first 6 months of treatment. Thus plaque control and salivary microbial colonization must be assessed after three and six months of treatment.

## ETHICS APPROVAL AND CONSENT TO PARTICIPATE

Ethics approval was obtained by the Ethic Committee of the University of L'Aquila, Italy (Document DR206/2013). The consent to the treatment was obtained by the patients before the beginning of the therapy.

## CONSENT FOR PUBLICATION

The consent to publish the present data was obtained from the subjects.

## AVAILABILITY OF DATA AND MATERIALS

The data that support the findings of this study are available from the archive of the University of L'Aquila, but restrictions apply to the availability of these data, which were used under permission and consent for the current study, and so are not publicly available. Data are however available from the authors upon reasonable request and with permission of the patients and the Ethic Committee of the University of L'Aquila.

## COMPETING INTERESTS

The authors declare that they have no competing interests

## FUNDING

This research did not receive any specific grant from funding agencies in the public, commercial, or not‐for‐profit sectors

## AUTHORS' CONTRIBUTIONS

SM: study conceiving, data recording, data analysis, data interpretation, manuscript writing

MT: data recording, data analysis, manuscript writing, data interpretation

AN: data analysis, manuscript writing, manuscript revision

SC: clinical activity, data recording

AD: manuscript writing, revisions

FA: clinical activity, data recording

GM: clinical activity supervision,

EM: manuscript writing supervision,

VQ: study conceiving, manuscript revision, study supervision, study coordination

ST: study conceiving, data handling, data analysis, data interpretation, manuscript writing

## References

[cre2261-bib-0001] Abbate, G. M. , Caria, M. P. , Montanari, P. , Mannu, C. , Orrù, G. , Caprioglio, A. , & Levrini, L. (2015). Periodontal health in teenagers treated with removable aligners and fixed orthodontic appliances. Journal of Orofacial Orthopedics, 76, 240–250. 10.1007/s00056-015-0285-5 25929710

[cre2261-bib-0002] Brignardello‐Petersen, R. (2019). Moderate‐to‐high levels of satisfaction and no important concerns in patients who received treatment with clear orthodontic aligners. Journal of the American Dental Association (1939), 150, e20 10.1016/j.adaj.2018.08.023 30290879

[cre2261-bib-0003] Caruso, S. , Nota, A. , Ehsani, S. , Maddalone, E. , Ojima, K. , & Tecco, S. (2019). Impact of molar teeth distalization with clear aligners on occlusal vertical dimension: a retrospective study. BMC Oral Health, 19, 182 10.1186/s12903-019-0880-8 31409348 PMC6692944

[cre2261-bib-0004] D'Ercole, S. , Martinelli, D. , & Tripodi, D. (2014). Influence of sport mouthguards on the ecological factors of the children oral cavity. BMC Oral Health, 14, 97 10.1186/1472-6831-14-97 25091394 PMC4146445

[cre2261-bib-0125] Dinoi, M. T. , Mummolo, S. , Monaco, A. , Marchetti, E. , Campanella, V. , & Marzo, G . (2015). Correction to: Orthodontic treatment of the transposition of a maxillary canine and a first premolar: A case report. Journal of Medical Case Reports, 9, 48 10.1186/s13256-015-0521-z 30832711 PMC6398243

[cre2261-bib-0005] Ferrazzano, G. F. , Scioscia, E. , Sateriale, D. , Pastore, G. , Colicchio, R. , Pagliuca, C. , … Pagliarulo, C. (2017). In Vitro Antibacterial Activity of Pomegranate Juice and Peel Extracts on Cariogenic Bacteria. BioMed Research International, 2017:2152749, 1–7. 10.1155/2017/2152749 PMC5676346 29209624

[cre2261-bib-0006] Freitas, A. O. A. , Marquezan, M. , Nojima, M. d. C. G. , Alviano, D. S. , & Maia, L. C. (2014). The influence of orthodontic fixed appliances on the oral microbiota: a systematic review. Dental Press Journal of Orthodontics, 19, 46–55. http://www.ncbi.nlm.nih.gov/pubmed/24945514 10.1590/2176-9451.19.2.046-055.oar PMC4296609 24945514

[cre2261-bib-0007] Giuca, M. R. , Pasini, M. , Tecco, S. , Giuca, G. , & Marzo, G. (2014). Levels of salivary immunoglobulins and periodontal evaluation in smoking patients. BMC Immunology, 15, 5 10.1186/1471-2172-15-5 24502245 PMC3918004

[cre2261-bib-0008] Kõll‐Klais, P. , Mändar, R. , Leibur, E. , & Kjaeldgaard, M. (2004). High levels of salivary lactobacilli in Estonian schoolchildren. European Journal of Paediatric Dentistry, 5, 107–109. http://www.ncbi.nlm.nih.gov/pubmed/15202924 15202924

[cre2261-bib-0009] Levrini, L. , Mangano, A. , Montanari, P. , Margherini, S. , Caprioglio, A. , & Abbate, G. M. (2015). Periodontal health status in patients treated with the Invisalign(®) system and fixed orthodontic appliances: A 3 months clinical and microbiological evaluation. European Journal of Dentistry, 9, 404–410. 10.4103/1305-7456.163218 26430371 PMC4569994

[cre2261-bib-0010] Lucchese, A. , Bondemark, L. , Marcolina, M. , & Manuelli, M. (2018). Changes in oral microbiota due to orthodontic appliances: a systematic review. Journal of Oral Microbiology, 10, 1476645 10.1080/20002297.2018.1476645 29988826 PMC6032020

[cre2261-bib-0011] Messer, L. B. (2000). Assessing caries risk in children. Australian Dental Journal, 45, 10–16. http://www.ncbi.nlm.nih.gov/pubmed/10846266 10846266 10.1111/j.1834-7819.2000.tb00235.x

[cre2261-bib-0012] Meuli, S. , Tecco, S. , Nota, A. , Gatto, R. , & Caruso, S. (2018). Clear aligners in pediatric age in a case of gingival recession due to malocclusion. Dental Cadmos, 86, 332 10.19256/d.cadmos.04.2018.09

[cre2261-bib-0013] Migliorati, M. , Isaia, L. , Cassaro, A. , Rivetti, A. , Silvestrini‐Biavati, F. , Gastaldo, L. , … Silvestrini‐Biavati, A. (2015). Efficacy of professional hygiene and prophylaxis on preventing plaque increase in orthodontic patients with multibracket appliances: a systematic review. European Journal of Orthodontics, 37, 297–307. 10.1093/ejo/cju044 25246605

[cre2261-bib-0126] Mummolo, S. , Nota, A. , Caruso, S. , Quinzi, V. , Marchetti, E. , & Marzo, G . (2018). Salivary markers and microbial flora in mouth breathing late adolescents. BioMed Research International, art. no. 8687608. 10.1155/2018/8687608 PMC5859862 29693018

[cre2261-bib-0127] Mummolo, S. , Nota, A. , De Felice, M. E. , Marcattili, D. , Tecco, S. , & Marzo, G (2018). Periodontal status of buccally and palatally impacted maxillary canines after surgical‐orthodontic treatment with open technique. Journal of Oral Science, 60(4), 552–556. 29984786 10.2334/josnusd.17-0394

[cre2261-bib-0014] Mummolo, S. , Marchetti, E. , Giuca, M. R. , Gallusi, G. , Tecco, S. , Gatto, R. , & Marzo, G. (2013). In‐office bacteria test for a microbial monitoring during the conventional and self‐ligating orthodontic treatment. Head & Face Medicine, 9, 7 10.1186/1746-160X-9-7 23375053 PMC3563582

[cre2261-bib-0015] Mummolo, S. , Tieri, M. , Tecco, S. , Mattei, A. , Albani, F. , Giuca, M. R. , & Marzo, G. (2014). Clinical evaluation of salivary indices and levels of Streptococcus mutans and Lactobacillus in patients treated with Occlus‐o‐Guide. European Journal of Paediatric Dentistry, 15, 367–370. http://www.ncbi.nlm.nih.gov/pubmed/25517581 25517581

[cre2261-bib-0016] Rossini, G. , Parrini, S. , Castroflorio, T. , Deregibus, A. , & Debernardi, C. L. (2015). Periodontal health during clear aligners treatment: a systematic review. European Journal of Orthodontics, 37, 539–543. 10.1093/ejo/cju083 25548145

[cre2261-bib-0017] Sánchez‐García, S. , Gutiérrez‐Venegas, G. , Juárez‐Cedillo, T. , Reyes‐Morales, H. , Solórzano‐Santos, F. , & García‐Peña, C. (2008). A simplified caries risk test in stimulated saliva from elderly patients. Gerodontology, 25, 26–33. 10.1111/j.1741-2358.2007.00184.x 18289130

[cre2261-bib-0018] Sifakakis, I. , Papaioannou, W. , Papadimitriou, A. , Kloukos, D. , Papageorgiou, S. N. , & Eliades, T. (2018). Salivary levels of cariogenic bacterial species during orthodontic treatment with thermoplastic aligners or fixed appliances: a prospective cohort study. Progress in Orthodontics, 19, 25 10.1186/s40510-018-0230-4 30066184 PMC6068060

[cre2261-bib-0019] Silvestrini‐Biavati, A. , Migliorati, M. , Demarziani, E. , Tecco, S. , Silvestrini‐Biavati, P. , Polimeni, A. , & Saccucci, M. (2013). Clinical association between teeth malocclusions, wrong posture and ocular convergence disorders: an epidemiological investigation on primary school children. BMC Pediatrics, 13(12). http://www.pubmedcentral.nih.gov/articlerender.fcgi?artid=3561080&tool=pmcentrez&rendertype=abstract, 10.1186/1471-2431-13-12 PMC3561080 23343244

[cre2261-bib-0021] Tecco, S. , Festa, F. , Salini, V. , Epifania, E. , & D'Attilio, M. (2004). Treatment of joint pain and joint noises associated with a recent TMJ internal derangement: A comparison of an anterior repositioning splint, a full‐arch maxillary stabilization splint, and an untreated control group. Cranio, 22, 209–219. 10.1179/crn.2004.026 15293777

